# Histological and clinical dose-response analysis of radiofrequency microneedling treatment for skin rejuvenation

**DOI:** 10.1007/s10103-025-04335-9

**Published:** 2025-02-07

**Authors:** Lynhda Nguyen, James Bartholomeusz, Stefan W. Schneider, Katharina Herberger

**Affiliations:** 1 Laser Department, Department of Dermatology and Venereology, University Medical Center Hamburg-Eppendorf, Martinistrasse 52, 20246 Hamburg, Germany; 2 Lutronic Medical Systems, 19 Fortune Drive, Billerica, MA 01821 USA; 3 SkinQRI, Lincolnshire, 19 Fortune Drive, Illinois, USA; 4 Department of Dermatology and Venereology, University Medical Center Hamburg-Eppendorf, Martinistrasse 52, 20246 Hamburg, Germany

**Keywords:** Radiofrequency microneedling, Insulated, Collagen coagulation, Coagulation volume, Skin rejuvenation

## Abstract

Radiofrequency microneedling (RFMN) is a commonly used fractional device to treat skin laxity and rhytids. Several studies investigated its histological and clinical effects. However, the role of the applied energy per needle (EPN) and total energy in the outcome remains unclear. The aim of the present study is to analyze the correlation between applied energy and resulting histological and clinical volume effects of RFMN treatment for skin rejuvenation. Ex vivo porcine skin was treated with a RFMN system equipped with insulated needles. Histological measurements of coagulation volumes were correlated with the delivered EPN, ranging from 20 mJ to 100 mJ. For the clinical investigation, a cohort of patients received treatment on the lower face and submental area. Absolute volume changes were calculated using computer-aided three-dimensional analysis. Pearson’s and Spearman’s correlation coefficients *r* were determined. Histologic analysis revealed a strong positive correlation between EPN and coagulation volume (*r* = 0.976; *p* < 0.005). A total of 30 patients, with a mean age of 55.9 ± 8.7 years, were recruited and received 1.7 ± 0.8 sessions (1–3) with a total energy of 1518.2 ± 784.1 J. Three-dimensional imaging revealed a strong logistic correlation with the total energy applied across all sessions (*r* = 0.676; *p* < 0.001). Analysis showed a positive association between the number of sessions and volume change. Study findings indicate a very strong and strong correlation between the applied energy and the resulting histological and clinical outcome in RFMN treatment, respectively. The introduction of coagulation volume as a matrix in RFMN treatments enables medical doctors to tailor and adjust treatment plan to the individual patient.

## Introduction

Fractional lasers and energy-based devices have become established modalities for addressing skin laxity and rhytids. Regardless of the specific system used, fractional devices are designed to create isolated zones of microthermal damage within the dermis while minimizing effects on surrounding tissue, thus promoting collagen remodeling and facilitating skin rejuvenation [1].

In the last decades, ablative-fractional lasers, such as carbon dioxide lasers, have become the gold standard in facial rejuvenation. However, their associated drawbacks, including extended downtime with inevitable adverse events like erythema, edema, and crust formation, and limitations for individuals with darker skin types, have driven the search for alternative treatment options [2, 3].

Radiofrequency microneedling (RFMN) has been recently introduced into the field of dermatology and has quickly emerged as a commonly used fractional device to enhance aging skin. In this technique, an array of needles serves as a delivery system for radiofrequency energy, targeting neocollagenesis and dermal volumization [4, 5]. Important parameters in RFMN treatment include insulated or non-insulated needles, needle depth, the energy per needle (EPN), and the applied total energy. A number of histological studies have aimed to characterize the thermal effects in the dermis after RFMN treatments [6–8]. These studies could provide characteristics of denatured collagen in the coagulation zones, along with structural changes in the surrounding tissue following RFMN treatment. In addition, Berube et al. found a positive temperature-lesion size curve using in vivo histology results [9].

Despite these histological investigations, a comprehensive understanding of the correlation between administered radiofrequency energy, coagulation volume, and associated clinical outcomes in skin rejuvenation, remains unclear. Therefore, the aim of the present study is to analyze the relationship between applied radiofrequency energy and resulting coagulation volumes and clinical volume changes in skin rejuvenation.

## Materials and methods

### Study design

For the present study, histological and clinical assessments were performed. The study was approved by the local ethics committees (PV7392) and conducted in accordance with the Good Clinical Practice and Declaration of Helsinki. The histological analysis of coagulation volumes was carried out using porcine samples. For clinical volumetric measurements, a cohort of patients received a RFMN treatment of their lower face and submental area at the dermatological laser department of the University Medical Center Hamburg-Eppendorf. Their clinical evaluation on the treatment has been published previously [10].

### Treatment protocol for histological evaluation

Abdominal skin from adult pigs was obtained from a local abattoir and utilized within 3 h post-mortem. The skin was treated with a RFMN system (Genius^®^, Lutronic Medical Systems) with energy per needle (EPN) settings ranging from 20 mJ to 100 mJ. A handpiece equipped with a 7 × 7 needle array was used to administer spot exposures, each covering an area of approximately 4 cm x 4 cm.

Tissue samples including the epidermis, dermis, and subcutis were collected, fixed in formalin, and examined using hematoxylin and eosin (H & E) staining. Volume of coagulation zones was measured using ImageJ software (National Institutes of Health, USA).

### Treatment protocol for clinical evaluation

The RFMN treatment protocol for the patients has been published previously [10]. After local anesthesia and thorough cleansing, patients received a RFMN treatment. Treatment parameters were selected based on the anatomical region and adjusted to pain sensitivity. The treatment involved three consecutive passes, alternating between horizontal and vertical directions. During the procedure, continuous air cooling (Cryo6, Zimmer Aesthetic Division) was administered to minimize discomfort. Patients received 1–3 treatment sessions at intervals of 4–12 weeks. The total energy applied during all treatment sessions on the lower face and submental regions was documented for further calculations. Control assessment was performed 3 and 6 months post-treatment.

### Standardized photo documentation and volume measurement

For standardized images, a three-dimensional camera system (Vectra^H2^, Canfield Scientific Inc.) with a white background and a single ring flashlight was used. Outer light sources were shielded. To maintain consistent head and neck positioning for each image, the bipupillar line and Frankfurt plane were set using a spirit level (PLL 1 P, Bosch^®^). Patients were instructed to keep their mouths closed and maintain a relaxed facial expression.

**Fig. 1:**
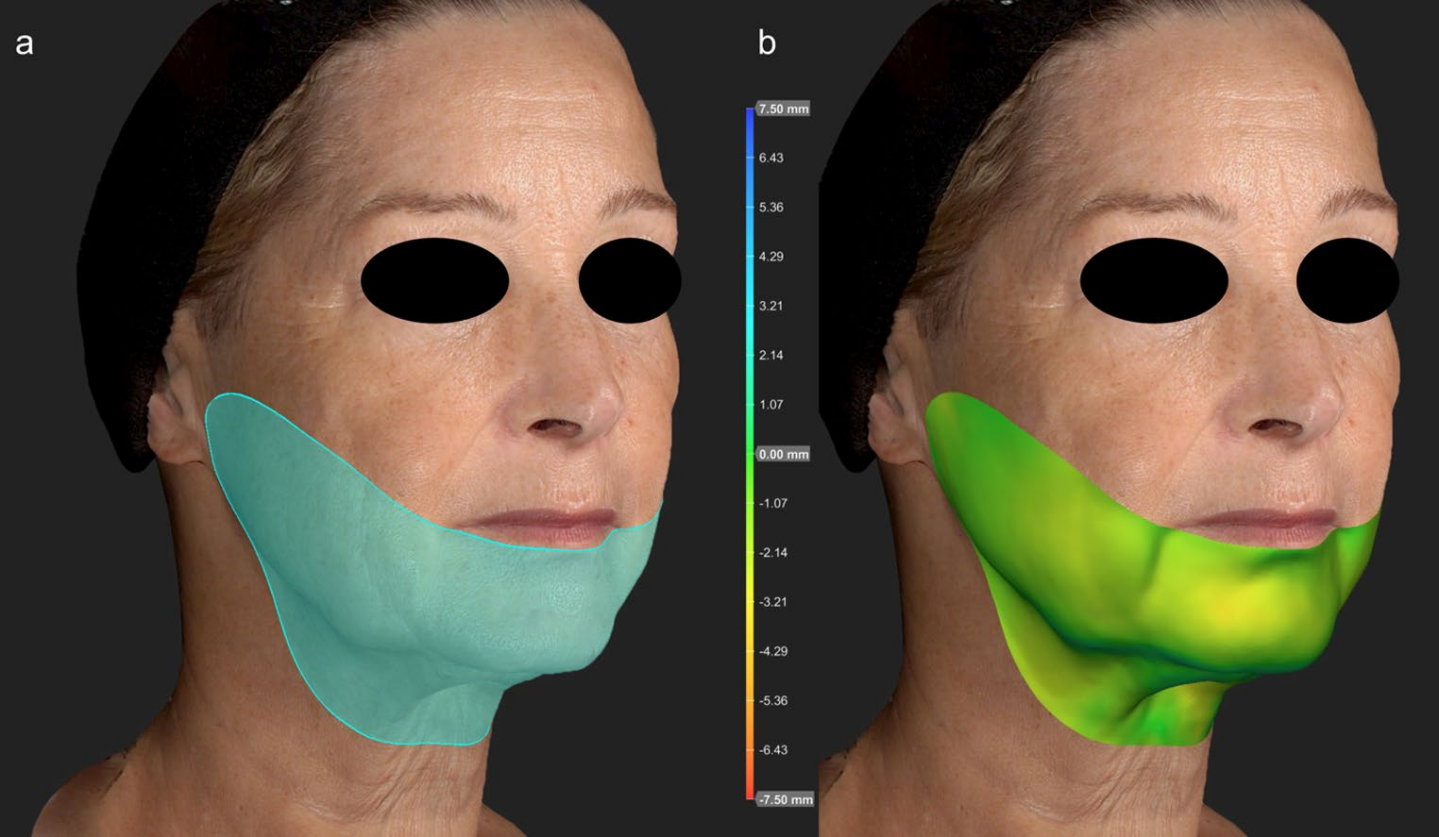
Computational three-dimensional analysis of volume change. **(a)** Selected lower face and submental area. **(b)** Color-coded volume difference six months post-treatment compared to baseline

One frontal and two 45° side photos were taken and then converted into a three-dimensional image using the Vectra Analysis Module software (VAM, Vectra^H2^, Canfield Scientific Inc.). To ensure accurate volume measurements, the positioning and orientation of the before and after images were precisely matched. The lower face and submental area were defined using following anatomical reference points: corners of the mouth, inferior margins of the external auditory meatus, mandibular angles, sternocleidomastoids, and laryngeal prominence. Automated analysis algorithms provided by the VAM software were employed to determine the absolute volume differences at the follow-up visit 6 months post-treatment compared to baseline (Fig. 1). Volume measurements were correlated with the total energy applied.

### Statistical analysis

Statistical analysis was performed using Microsoft^®^ Excel (Microsoft Corporation, Version 16.77.1) and GraphPad Prism (GraphPad software, Version 10). The Pearson’s and Spearman’s correlation coefficient *r* was used to calculate the strength of association between linear and non-linear variables, respectively. If not stated otherwise, descriptive data were presented as means ± standard deviation, ranges (minimum– maximum), and 95% confidence interval (CI). P-values < 0.05 were defined as significant.

## Results

### Histologic analysis

**Fig. 2:**
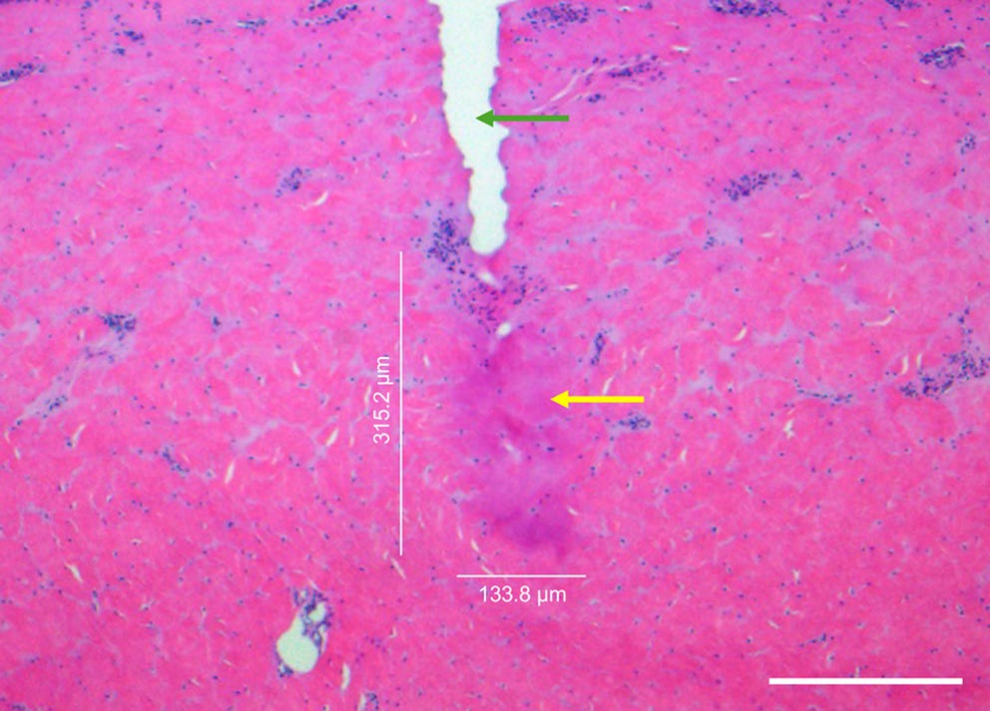
Cross-sectional histological image immediately after radiofrequency microneedling (40 mJ per needle, 2.2 mm needle depth). The coagulation volume appeared clearly in the dermal layer and was measured to be 0.047 mm^3^. Green arrow indicates area of needle penetration. Yellow arrow indicates coagulation zone. Height and width of the coagulation area are labeled. Scale bar: 200 μm

**Fig. 3:**
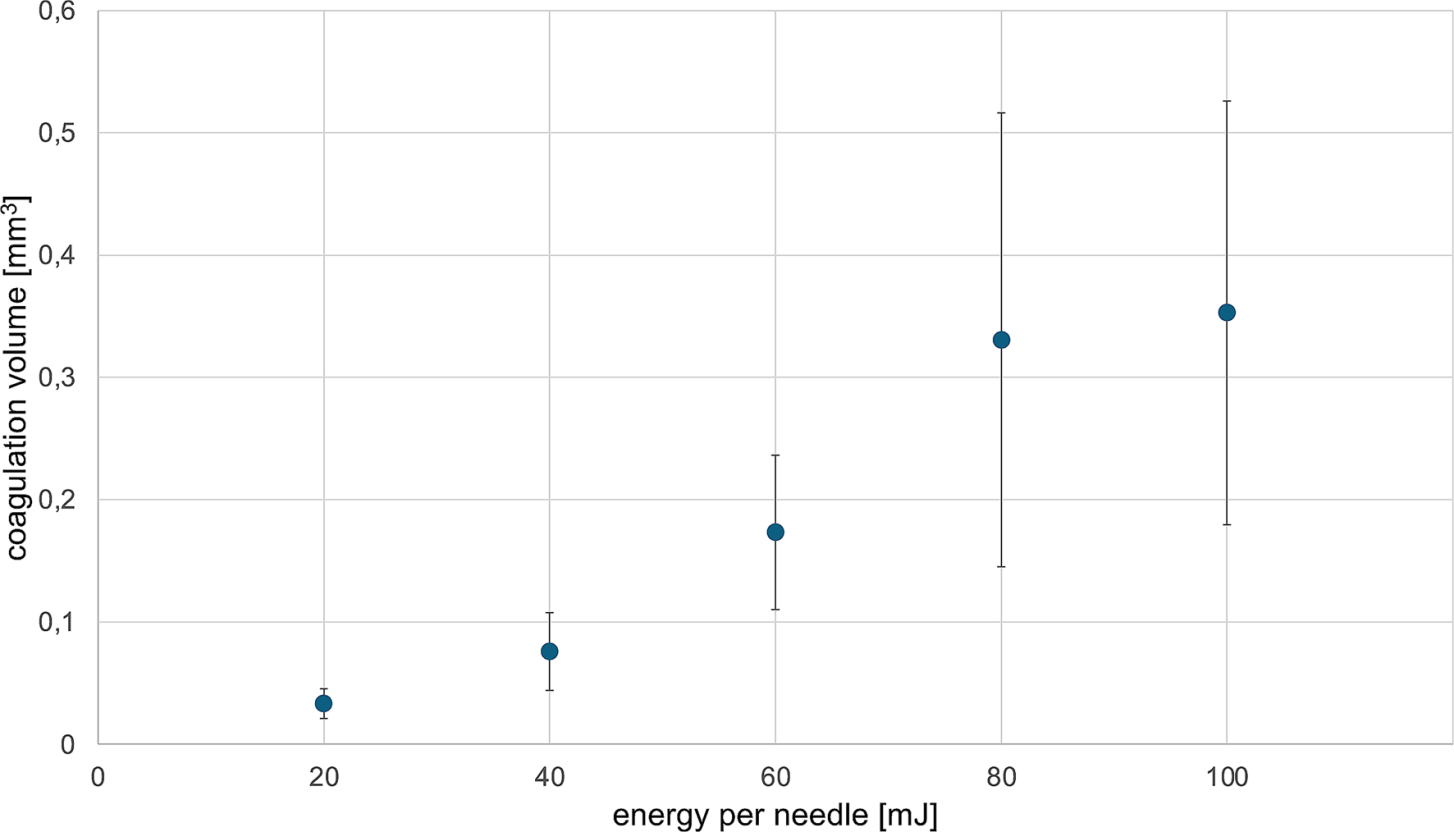
Histological analysis of porcine skin revealed a positive correlation between energy per needle and coagulation volume after radiofrequency microneedling treatment

**Fig. 4:**
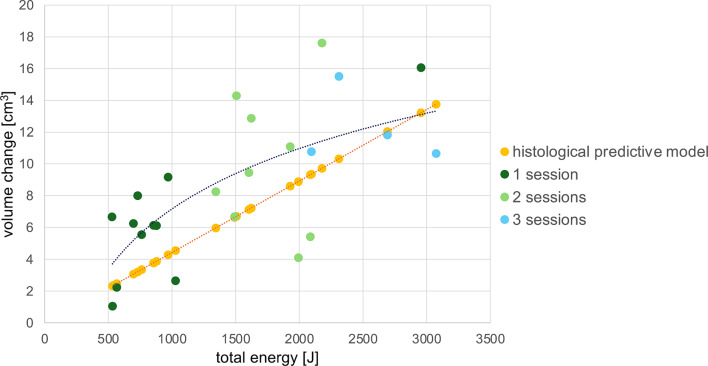
Logistic correlation of total volume change of the lower face and submental area and total energy applied during radiofrequency microneedling treatment depending on the number of sessions. Orange line: linear correlation model based on ex vivo histological analysis. Blue line: clinical analysis based on three-dimensional images

Porcine skin samples from ten different specimens were used. For each EPN level, coagulation volumes were measured in five areas per sample, resulting in a total of 50 coagulation volume measurements for each EPN level. Zones with denatured collagen within the dermal layer were clearly demarcated from surrounding intact tissue (Fig. 2). These coagulation zones exhibited an ellipsoidal shape, prompting further investigation into their volumetric properties. At an EPN of 20 mJ, 40 mJ, 60 mJ, 80 mJ, and 100 mJ coagulation volume increased to 0.033 ± 0.012 mm^3^, 0.076 ± 0.032 mm^3^, 0.173 ± 0.063 mm [3], 0.288 ± 0.320 mm [3], and 0.353 ± 0.173 mm^3^, respectively. While a positive correlation between the ellipsoidal coagulation volumes and the EPN could be identified, there were notable deviations in volume measurements (Fig. 3). The Pearson’s correlation coefficient *r* was 0.976 (95% CI: 0.659–0.998; *p* < 0.05). Based on these ex vivo observations and assuming a nearly linear correlation, we formulated a predictive model depicting the absolute volume change as a function of the total applied energy (Fig. 4).

## Baseline characteristics of patients for the clinical assessment

In total, 30 patients with a mean age of 55.9 ± 8.7 years (38–75) were recruited. Six patients were excluded from volume analysis: one lost to follow-up, two with > 5% body weight difference, two with inconsistent head and neck positioning, and one with hair in the region of interest making volume analysis not feasible. Patients received a mean number of 1.7 ± 0.8 sessions (1–3) with a total energy of 1518.2 ± 784.1 J (532.4–3075.5).

### Clinical correlation of volume difference and total energy

Volumetric analysis of the lower face and submental area was performed using computer-aided analysis, revealing a logistic correlation with the applied total energy. Patients were categorized based on the number of treatment sessions they underwent. Except for one patient, they showed a positive association between the number of sessions and increased volume change (Fig. 4). A Spearman’s correlation coefficient *r* = 0.676 (95% CI: 0.363–0.852) with a p-value of < 0.001 was calculated.

## Discussion

This study conducted an analysis of coagulation volume and clinical outcomes, in correlation with the applied radiofrequency energy. It establishes the first evidence on the positive correlation of coagulation volume, clinical outcomes, and applied energy through ex vivo histological and clinical three-dimensional imaging analysis.

In RFMN treatment, microneedles deliver thermal energy into the dermis. Depending on the system used, the applied energy results in fractional tissue coagulation or ablation. The subsequent wound healing process of the dermal collagen induces its regeneration and neocollagenesis, leading to skin tightening and improvement of skin laxity [6, 11, 12]. Insulated microneedles only deliver energy through the tip of the needle, thereby minimizing the risk of epidermal damage.

The relationship between temperature and coagulation is a critical factor in RFMN treatment. In this study, we employed an insulated RFMN system with real-time feedback mechanism to minimize the risk of overheating and unwanted tissue responses. The device was set to automatically adjust pulse duration, which increased with higher EPN levels. Several studies investigated the ideal settings for skin rejuvenation. Overly aggressive treatments, i.e. high temperatures, long delivery durations, and closely spaced treatments, resulted in less clinical improvement [13–16]. According to these studies, temperatures at 67 °C, 3 to 4 mm spacing between needle penetration, and needle lengths equivalent to the reticular dermis result in an optimal clinical outcome [15].

Based on these data, we hypothesize that the applied total energy directly correlates with the coagulation volume and subsequent clinical outcome in RFMN treatment for skin rejuvenation. While previous studies have investigated histological characteristics and ideal parameters, to our best of knowledge, a correlation analysis that combines both histology and clinical volume measurements have not been published to date.

Through ex vivo investigation, we found a strong positive correlation between coagulation volume and delivered EPN. Based on these data, we formulated a positive correlation model between clinical volume difference and applied total energy. Considering the nature of ex vivo histology and the sample shrinkage caused by fixation, it is important to note that the actual coagulation volume might be larger than the measured values. In contrast, our clinical evaluation, conducted through computer-aided three-dimensional analysis, revealed a logistic correlation in volume change with increasing energy. This is in line with the limited capacity of the skin to change volume through neocollagenesis. Moreover, the clinical response is influenced by intrinsic factors, such as age, skin quality, and genetics. Extrinsic factors like UV exposure, smoking, nutrition, and stress may also impact the outcomes. Our analysis also indicated a positive association between advanced age and clinical response. Due to the sample size, a valid sub-analysis could not be conducted.

In this study, the calculation model is based under the assumption that alterations in volume - whether a decrease or increase - are based on the principle of neocollagenesis. Clinical evidence in RFMN treatments has demonstrated both volume reductions in the jawline region and volume increases in the midface area, associated with skin thickening. Consequently, the clinical role of collagen remodeling extends beyond mere skin tightening, introducing complexity to the calculations. Nonetheless, our data provides valuable insights for evaluating the effects and planning treatments.

For planning treatments with laser and energy-based devices, a comprehensive understanding of their effects is necessary. Considering coagulation volume as a matrix in RFMN treatment is important for anticipating clinical outcomes, ensuring patient safety, and tailoring treatments to achieve desired results. This approach to volume measurement can also be applied to other fractional technologies that aim for skin rejuvenation through collagen remodeling.

Our histological analysis showed a high standard deviation of volume measurement. This may be because the histology slices were not consistently positioned at the center of the coagulation zone or parallel to the needle puncture. Enhancing the representativeness of the image set for processing could potentially improve the positive relationship between EPN and coagulation volume. In the clinical analysis, inconsistencies in treatment, such as overlapping shots, and variations in photo documentation, despite intended standardization measures, may introduce additional deviations in measurements besides the intrinsic and extrinsic influencing factors.

This study has some limitations. Firstly, our microscopic investigations were applied to ex vivo porcine abdominal skin, which may restrict the generalizability of the results. Additionally, we could not eliminate the potential influence of patients’ lifestyles, e.g. UV exposure and diet. Moreover, due to the sample size, conducting a sub-analysis to determine the impact of age and skin quality to the clinical outcome following RFMN treatment was not feasible.

In conclusion, our study indicates a strong positive correlation between the applied energy and the resulting histological and clinical outcome in RFMN treatments. The introduction of coagulation volume as a matrix in RFMN treatments represents a paradigm shift, offering a new perspective on treatment planning. However, to refine our understanding and approach in RFMN treatments, additional prospective clinical studies are warranted. In particular, the influence of the number and interval between treatment sessions has not been clarified yet, nor have the long-term effects. In our study, treatment intervals were extended to three months instead of the recommended four weeks by the manufacturer. This decision was based on our observations of the duration of clinical changes resulting from neocollagenesis. Although the optimal interval remains scientifically unclear, addressing this question thoroughly is crucial for providing practical and well-founded recommendations. Additionally, the correlation between pulse duration and treatment outcome warrants further investigation. Future studies should delve into in-depth sub-analyses to explore how individual patient characteristics influence treatment outcomes and guide the formulation of individualized treatment plans.

## Data Availability

The data that support the findings of this study are available from the corresponding author upon reasonable request.

## References

[1] Anderson RR, Parrish JA (1983) Selective photothermolysis: precise microsurgery by selective absorption of pulsed radiation. Science 220(4596):524–527 6836297 10.1126/science.6836297

[2] Chen SX, Cheng J, Watchmaker J, Dover JS, Chung HJ (2022) Review of lasers and energy-based devices for skin rejuvenation and scar treatment with histologic correlations. Dermatol Surg 48(4):441–448 35165220 10.1097/DSS.0000000000003397

[3] El-Domyati M, Abd-El-Raheem T, Abdel-Wahab H, Medhat W, Hosam W, El-Fakahany H et al (2013) Fractional versus ablative erbium:yttrium-aluminum-garnet laser resurfacing for facial rejuvenation: an objective evaluation. J Am Acad Dermatol 68(1):103–112 23110966 10.1016/j.jaad.2012.09.014

[4] Kneiber D, Amin M, Nguyen TA, Gharavi NM (2023) Review of radiofrequency microneedling: history, devices and uses. J Cosmet Laser Ther, 25(5–8):59–64 10.1080/14764172.2023.2268308 37844087

[5] Lim SD, Yeo UC, Kim IH, Choi CW, Kim WS (2013) Surgical corner. Evaluation of the wound healing response after deep dermal heating by fractional micro-needle radiofrequency device. J Drugs Dermatol 12(9):1044–1049 24002154

[6] Hantash BM, Renton B, Berkowitz RL, Stridde BC, Newman J (2009) Pilot clinical study of a novel minimally invasive bipolar microneedle radiofrequency device. Lasers Surg Med 41(2):87–95 19226570 10.1002/lsm.20687

[7] Cho SB, Na J, Zheng Z, Lim JM, Kang JS, Lee JH et al (2018) In vivo skin reactions from pulsed-type, bipolar, alternating current radiofrequency treatment using invasive noninsulated electrodes. Skin Res Technol 24(2):318–325 29368439 10.1111/srt.12433

[8] Feng J, Zhang L, Qi J, Huang L (2023) Histological damage characteristics and quantitive analysis of porcine skin with non-insulated microneedle radiofrequency. Skin Res Technol 29(6):e13396 37357651 10.1111/srt.13396 PMC10264746

[9] Berube D, Renton B, Hantash BM (2009) A predictive model of minimally invasive bipolar fractional radiofrequency skin treatment. Lasers Surg Medicine: Official J Am Soc Laser Med Surg 41(7):473–478 10.1002/lsm.20794 19708063

[10] Nguyen L, Blessmann M, Schneider SW, Herberger K (2022) Radiofrequency Microneedling for skin tightening of the Lower Face, Jawline, and Neck Region. Dermatol Surg 48(12):1299–1305 36449871 10.1097/DSS.0000000000003607

[11] Tanaka Y (2015) Long-term three-dimensional volumetric assessment of skin tightening using a sharply tapered non-insulated microneedle radiofrequency applicator with novel fractionated pulse mode in asians. Lasers Surg Med 47(8):626–633 26272454 10.1002/lsm.22401

[12] Kim KE, Park JH, Seul TW, Kim IH, Ryu HJ (2023) Periorbital skin rejuvenation of Asian skin using Microneedle Fractional Radiofrequency. Ann Dermatol 35(5):360–366 37830418 10.5021/ad.22.217 PMC10579575

[13] Alexiades-Armenakas M, Newman J, Willey A, Kilmer S, Goldberg D, Garden J et al (2013) Prospective multicenter clinical trial of a minimally invasive temperature-controlled bipolar fractional radiofrequency system for rhytid and laxity treatment. Dermatol Surg 39(2):263–273 23278964 10.1111/dsu.12065

[14] Alexiades M, Berube D (2015) Randomized, blinded, 3-arm clinical trial assessing optimal temperature and duration for treatment with minimally invasive fractional radiofrequency. Dermatol Surg 41(5):623–632 25915628 10.1097/DSS.0000000000000347

[15] Tan M, Jo C, Chapas A, Khetarpal S, Dover J, Tan MG et al (2021) Radiofrequency Microneedling: a Comprehensive and critical review. Dermatol Surg 47(6):755–761 33577211 10.1097/DSS.0000000000002972

[16] Lu W, Wu P, Zhang Z, Chen J, Chen X, Ewelina B (2017) Curative effects of microneedle fractional radiofrequency system on skin laxity in Asian patients: a prospective, double-blind, randomized, controlled face-split study. J Cosmet Laser Therapy 19(2):83–88 10.1080/14764172.2016.1256485 27849406

